# Age-Related Midbrain Inflammation and Senescence in Parkinson’s Disease

**DOI:** 10.3389/fnagi.2022.917797

**Published:** 2022-06-02

**Authors:** Taylor Russo, Markus Riessland

**Affiliations:** ^1^Department of Neurobiology and Behavior, Stony Brook University, Stony Brook, NY, United States; ^2^Center for Nervous System Disorders, Stony Brook University, Stony Brook, NY, United States

**Keywords:** Parkinson’s disease, cellular senescence, dopamine neurons, immune response, neuroinflammation, aging

## Abstract

Immune responses are arising as a common feature of several neurodegenerative diseases, such as Parkinson’s disease (PD), Alzheimer’s disease (AD), and Amyotrophic Lateral Sclerosis (ALS), but their role as either causative or consequential remains debated. It is evident that there is local inflammation in the midbrain in PD patients even before symptom onset, but the underlying mechanisms remain elusive. In this mini-review, we discuss this midbrain inflammation in the context of PD and argue that cellular senescence may be the cause for this immune response. We postulate that to unravel the relationship between inflammation and senescence in PD, it is crucial to first understand the potential causative roles of various cell types of the midbrain and determine how the possible paracrine spreading of senescence between them may lead to observed local immune responses. We hypothesize that secretion of pro-inflammatory factors by senescent cells in the midbrain triggers neuroinflammation resulting in immune cell-mediated killing of midbrain dopaminergic (DA) neurons in PD.

## Introduction

Parkinson’s disease (PD) is an age-related disorder that is characterized by the progressive loss of dopaminergic (DA) neurons in the substantia nigra pars compacta (SNpc) of the midbrain, which results in well-characterized motor symptoms (Bernheimer et al., 1973; Fearnley and Lees, 1991; de Rijk et al., 2000; Strickland and Bertoni, 2004). In addition to age, PD risk factors include environmental toxins, drugs, and pesticides, and genomic mutations (Emamzadeh and Surguchov, 2018). Although the cause of sporadic PD is unknown, it has been hypothesized that pre-symptomatic midbrain inflammation plays a critical role (Riessland, 2020). DA neurons of the SNpc are particularly vulnerable because of their high levels of reactive dopamine, high energy demand and mitochondrial turnover, calcium handling, and their large axonal arborizations (Guzman et al., 2010; Pacelli et al., 2015; Riessland et al., 2017). Recently, it has been shown that these neurons are particularly prone to enter a state of cellular senescence (Riessland et al., 2019). In line with this discovery, it has been reported that both mitotic and post-mitotic cells in the brain have the potential to become senescent, including neurons, astrocytes, microglia, endothelial cells, and oligodendrocyte progenitor cells (Minamino et al., 2002; Streit et al., 2004; Bitto et al., 2010; Jurk et al., 2012; Zhang et al., 2019). Cellular senescence can be described as a multi-step evolution, with an initial transition into stable cell-cycle arrest characterized by prolonged p21 and/or p16 activity. Activation of p16 or p21 is usually driven by activation of the DNA damage response machinery (Yoon et al., 2022). Progression into a full state of senescence includes lamin B1 downregulation, triggering both local and global modifications in chromatin methylation (Hayflick and Moorhead, 1961; Kurz et al., 2000; Beauséjour et al., 2003; Shimi et al., 2011; van Deursen, 2014). Additional phenotypes of senescent cells include the senescence-associated secretory phenotype (SASP), dysfunctional mitochondria and lysosomes, elevated levels of reactive oxygen species (ROS), lipofuscin accumulation, and senescence-associated β-galactosidase (SA-β-gal) (Martínez-Zamudio et al., 2017). These features are characteristic of both mitotic and post-mitotic senescent cells and their combined presence is widely used for defining senescent cells *in vitro* and *in vivo*. The SASP involves the secretion of growth factors, chemokines, and cytokines into the extracellular space acting to modify the microenvironment, trigger immune surveillance, and mediate a paracrine transmission of senescence (Rodier and Campisi, 2011; Acosta et al., 2013; Tasdemir and Lowe, 2013; Hohn et al., 2017). With age, there is evidence of a declined immune response to senescence where these senescent cells are less efficiently removed by the adaptive and innate immune systems (van Deursen, 2014). Interestingly, there is increasing evidence that the adaptive and innate immune systems are involved in the progression of PD. Activated microglia directly contribute to the loss of DA neurons in the midbrain in PD (Qian and Flood, 2008) and activated cells of the adaptive immune system are present in postmortem PD brain tissues (Garretti et al., 2019). Although brain senescence in PD is evident, it remains unclear how the cellular senescence of DA neurons relates to the inflammation seen in PD. We hypothesize that the cellular senescence of DA neurons itself is the cause of the local inflammation in the midbrain observed in PD. In order to better understand the relationship between inflammation and cellular senescence, it is important to characterize the various cell types at play and their potential role in the neuroinflammation observed in the midbrain of PD patients.

## Senescence Associated Secretory Phenotype

Senescence associated secretory phenotype (SASP) expression by senescent cells reinforces senescence, activates immune surveillance, and has pro-tumorigenic properties (Acosta et al., 2013). This phenotype of senescent cells is characterized by the secretion of soluble factors such as interleukin-6 (IL-6), interleukin-1 (IL-1), and chemokine ligand 1 (CXCL1), secreted proteases including matrix metalloproteinases (MMPs), and secreted insoluble proteins such as extracellular matrix components (Coppé et al., 2010; Acosta et al., 2013). Interestingly, proinflammatory cytokines are elevated in the blood of PD patients and their levels correlate with clinical stage of the disease (Garretti et al., 2019). The SASP develops dynamically over time and has been shown to promote cell proliferation, stimulate cell motility, regulate cell differentiation, and affect leukocyte infiltration, causing inflammation. In addition, the SASP has been shown to induce a paracrine spreading of senescence in normal cells both in culture and in mouse models of oncogene induced senescence (OIS) *in vivo* (Acosta et al., 2013). There are multiple SASP components that are known to mediate this paracrine senescence, including vascular endothelial growth factor (VEGF), chemokine ligand 2 (CCL2), and chemokine ligand 20 (CCL20). Expression of the SASP is controlled by inflammasome-mediated IL-1 signaling, which is activated in senescent cells (Acosta et al., 2013). Other SASP factors are more closely associated with the reinforcement of senescence, including insulin like growth factor binding protein 7 (IGFBP-7), plasminogen activator inhibitor 1 (PAI-1), IL-6, and chemokine receptor 2 (CXCR2)-binding chemokines such as interleukin-8 (IL-8) or CXCL1. The SASP contributes to the surveillance and elimination of senescent cells by the immune system (Acosta et al., 2013). It has been shown that p21, a potent cyclin-dependent kinase inhibitor that regulates cell proliferation, plays an important role in SASP expression which can induce immunosurveillance of senescent cells to recruit macrophages as a first line of defense (Sturmlechner et al., 2021). In terms of PD, this could play a role in loss of DA neurons in the SNpc. Based on the upregulation of CCL2, interleukin-17 receptor (IL17-R), and major histocompatibility complex (MHC) genes human leukocyte antigen B (HLA-B) and C (HLA-C) in senescent midbrain DA neurons (Riessland, 2020), it is plausible that the SASP of senescent midbrain DA neurons in PD patients triggers both an adaptive and innate immune response. Specifically, CCL2 has been shown to attract T-cells which actively kill DA neurons that express the IL17-R in PD patients via the interleukin-17 (IL-17) pathway (Garretti et al., 2019).

## Neurons

In contrast to the classic view of senescence as an irreversible cell-cycle arrest mechanism, senescence in post-mitotic neurons has become evident (Jurk et al., 2012; van Deursen, 2014). The production of proinflammatory cytokines and chemokines are a common feature of senescent cells, regardless of the stressor or mechanism that induces the senescence (van Deursen, 2014). The SNpc of the midbrain contains DA neurons which have been shown to become senescent in PD (Riessland et al., 2019) and have been characterized to express various markers of cellular senescence. This neuronal senescence can be mediated by the loss of transcription factor and PD risk factor *special AT-rich sequence-binding protein 1* (SATB1), which binds to and represses *CDKN1A*, the gene encoding p21 protein in DA neurons. Neurons in the PD-vulnerable brain region the SNpc express high levels of SATB1, whereas ablation of SATB1 (SATB1-KO) was shown to induce neuronal senescence in midbrain DA neurons (Riessland et al., 2019). Elimination of SATB1 in cortical neurons did not induce senescence, suggesting a cell type-specific role of SATB1. Accordingly, removal of p21 was shown to robustly reduce inflammation (Jurk et al., 2012), suggesting that neuronal senescence may contribute to the ‘inflamm-aging’ seen in PD. SATB1-knockdown *in vivo* led to p21 elevation and consequently to a local immune response (Riessland et al., 2019). Interestingly, specific sub-types of neurons have also been found to enter a state of cellular senescence in Alzheimer’s disease (AD) (Musi et al., 2018; Dehkordi et al., 2021).

## Microglia

Microglia are glial cells that function as macrophages in the central nervous system as a part of the innate immune response (Yang et al., 2010). In the normal aging brain there is a constant, low level of chronic inflammation which has been termed ‘inflamm-aging’ (Franceschi et al., 2000; Chinta et al., 2015). Chronic inflammation is characterized by the production of cytokines and has been shown to act as both an intrinsic and extrinsic inducer of senescent phenotypes in microglia (Franceschi et al., 1999; Yu et al., 2012; Caldeira et al., 2014; Sah et al., 2021). With age, there is evidence for age-associated microglia senescence which includes both morphological changes and an inflammatory profile shift (Luo et al., 2010). The production of inflammatory products by microglia has been shown to contribute to DA neuron death in PD (Qian and Flood, 2008). There is evidence for an overactivation of microglia in PD patients, which could point to microglia being primed to become neurotoxic (Luo et al., 2010). Lipopolysaccharide (LPS) injections, which are widely used to stimulate an immune response, have been shown to lead specifically to SNpc neurodegeneration with a loss of DA neurons. This LPS-induced neurotoxicity and region-specific DA neuron susceptibility was positively correlated with microglia density, which was highest in the SNpc (Kim et al., 2000). α-Synuclein accumulation has been shown to lead to a robust inflammatory activation of microglia (Grozdanov et al., 2019) and interaction between microglia and α-synuclein has been suggested to play a role in the propagation of α-synuclein aggregation in PD (Sanchez-Guajardo et al., 2013; Tansey et al., 2022). Additionally, SATB1-knockdown senescent midbrain DA neurons, as a model of *in vivo* senescent cells, were shown to be actively removed by microglia (Riessland et al., 2019).

## Astrocytes

In addition to microglia, astrocyte senescence in aging has been clearly demonstrated (Pertusa et al., 2007; Bitto et al., 2010; Chinta et al., 2015). Specifically, senescent astrocytes have been found in PD brain tissue (Chinta et al., 2018). In response to treatment with paraquat (PQ), a well-known environmental toxin that has been correlated with increased risk for developing PD, astrocyte senescence was shown to be induced *in vitro* and *in vivo*. This astrocyte senescence included a SASP and depletion of these senescent cells protected against the PQ-induced neuropathology (Chinta et al., 2018). Further support for a role of astrocyte senescence in neurodegeneration comes from AD research, where senescent astrocytes have been observed in AD and have been shown to be triggered by β-amyloid and to produce inflammatory cytokines (Bhat et al., 2012).

## Oligodendrocytes

Cellular senescence is apparent in astrocytes, microglia, and neurons of the midbrain which are neighbors to oligodendrocytes. There is accumulating evidence of senescence-like features in oligodendrocytes and their progenitors (Sams, 2021) with increasing age, but a clear demonstration of oligodendrocyte senescence in PD has yet to be shown. Senescence of oligodendrocytes has been reported in white matter lesions which are common in brain aging and are associated with dementia (Al-Mashhadi et al., 2015). Oligodendrocyte progenitor cells exhibit a senescence-like phenotype characterized by upregulated p21, p16, and SA-β-gal activity in AD mouse models as well as in the brains of patients with AD (Zhang et al., 2019).

## Endothelial Cells

Endothelial cell senescence is evident in the aging and diseased brain (Nation et al., 2019; Graves and Baker, 2020), contributing to an age-dependent uncoupling of the neurovascular unit and impairment of the integrity of the blood brain barrier (BBB). This leads to increased permeability of the BBB, which results in neurotoxicity in both aging and disease. Senescent endothelial cell accumulation leads to increase SASP expression, which in turn could stimulate chronic neuroinflammation (Graves and Baker, 2020). This endothelial cell senescence at the BBB has been shown to directly contribute to BBB breakdown (Yamazaki et al., 2016). In line with the development of age-related neurodegenerative diseases, the BBB has been shown to be compromised in the midbrain region of PD patients (Garretti et al., 2019). It is thus plausible that in PD, endothelial cells at the BBB become senescent causing impairment of BBB integrity allowing for the paracrine spreading of senescence via the SASP to diverse cell types in the midbrain or for the SASP of BBB endothelial cells itself to trigger an immune response.

## Pericytes

Pericytes act to maintain the BBB and regulate immune cell entry into the central nervous system (Attwell et al., 2016). Although the existence of senescent pericytes in PD is not known, treatment with hydrogen peroxide, a commonly used model for stress-induced senescence, led to senescence in pericytes as shown by a decreased growth rate and increased p16 levels (Yamazaki et al., 2016).

## Peripheral Immune Cells (T-Cells)

Inflammation is a characteristic hallmark of many neurodegenerative disorders, including AD, ALS, multiple sclerosis (MS), and PD (Baker et al., 2011; Chitnis and Weiner, 2017; Trias et al., 2019) and there is evidence that the immune cells play an active role. Aging and age-related neurodegenerative diseases are associated with increased SASP-expressing senescent cells of non-neuronal origin in the brain and this correlates with the degree of neurodegeneration (Chinta et al., 2015). A link between neuroinflammation and PD pathogenesis was made apparent with the discovery of reactive microglia positive for human leukocyte antigen-DR isotype (HLA-DR), a marker of T-cell activation, in the SNpc of PD patients (McGeer et al., 1988). It was later shown that the number of HLA-DR+ microglia correlates with the degree of neurodegeneration along the nigrostriatal pathway (Imamura et al., 2003; Tansey et al., 2022). The exact role of the adaptive immune system in PD pathogenesis is unclear, although there is increasing evidence that it is indeed involved. It is hypothesized that this SASP recruits natural killer cells to facilitate the removal of senescent cells and neighboring tumor cells. DA receptors are expressed by both cluster of differentiation 4 (CD4)+ and 8 (CD8)+ T-cells (Baird et al., 2019; Liu et al., 2021) and CD4+ and CD8+ T-cells have been shown to infiltrate the midbrain in animal models of PD (Galiano-Landeira et al., 2020). In an animal model for PD treated with 6-hydroxydopamine (6-OHDA), which is a well-characterized neurotoxin that specifically targets DA neurons, T-cell infiltration was observed (Baird et al., 2019). In an assessment of T-cell infiltration in the brain of PD patients, a ten-fold increase in both CD4+ and CD8+ T-cells was determined (Brochard et al., 2009). CD8+ T-cells were seen in the SNpc of PD patients both surrounding and contacting DA neurons (Galiano-Landeira et al., 2020). A higher density of CD8+ T-cells was correlated with a lower density of DA neurons in PD patients, indicating that CD8+ T-cells are key mediators of neuronal death in PD. This CD8+ T-cell infiltration preceded both α-synuclein aggregation and neuronal cell death, where patients with incidental Lewy body disease (iLBD), which is considered to be an early, pre-symptomatic stage of PD, also demonstrated CD8+ T-cell infiltration in the SNpc and colocalization with midbrain DA neurons. This indicates an adaptive immune attack on midbrain neurons before neurodegeneration or protein aggregation were apparent (Galiano-Landeira et al., 2020). Thus, CD8+ T-cells contribute to nigral DA neuron impairment and death in PD even before Lewy body deposition. T-cells observed in post-mortem PD brain tissue have been shown to actively kill midbrain DA neurons via the IL-17 pathway in sporadic PD cases (Garretti et al., 2019). In addition, DA neurons are known to express MHC-I under control conditions (Cebrián et al., 2014). In PD specifically, DA neurons have been shown to express MHC which present α-synuclein as an antigen causing T-cell activation in patients (Sulzer et al., 2017). Elevated α-synuclein T-cell responses have been reported prior to PD diagnosis and α-synuclein reactive T-cells are present in PD patients (Lindestam Arlehamn et al., 2020). It is specifically the increased presence of CD8+ T-cells in the midbrain leading to dysregulation associated with the severity of PD, rather than patient age, age of onset, or the duration or progression of PD (Bhatia et al., 2021). Since we have found that senescent DA neurons upregulate HLA-B and HLA-C, it is likely that these cells are highly prone to be attacked by killer T-cells (Riessland et al., 2019; Riessland, 2020). It is plausible that the SASP expressed by senescent DA neurons would trigger a long-distance attraction, recruiting and activating T-cells prior to their attack.

## Discussion

**FIGURE 1:**
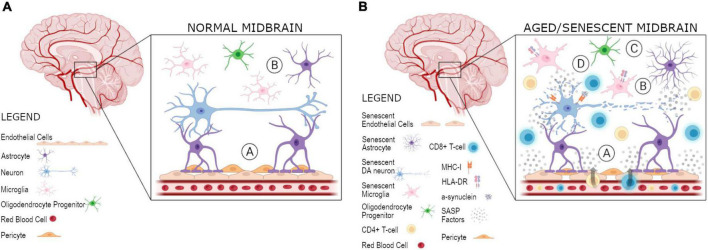
Proposed model for spreading of senescence and local inflammation in the midbrain. Various cell types, including DA neurons, microglia, astrocytes, and OPCs, are present in the midbrain. **(A)** At the BBB there is a coupling of the neurovascular unit in a normal midbrain, composed of the brain vasulature containing endothelial cells, as well as astrocytes, and their connections to neurons: (A) an impermeable BBB of normal integrity is present and (B) microglia and astrocytes are present for surveillance and support of DA neurons, respectively. **(B)** In an aged/senescent midbrain, there are three plausible hypotheses for the spreading of cellular senescence and its role in the uncoupling of the neurovascular unit and local inflammation: (A) endothelial cells at the BBB become senescent and release SASP factors, resutling in a recruitment of T-cells, which are able to enter the midbrain as a result of weakened BBB integrity, (B) midbrain DA neurons become senescent in PD and their secreted SASP factors are able to spread senescence to other cell types, including astrocytes, microglia, OPCs, and/or endothelial cells, which eventually leads to the weakening of the BBB and recruitment of adaptive immune cells, or (C) some other cell type in the midbrain, astrocytes, microglia, OPCs, or endothelial cells, become senescent and act negatively on surrounding cells by initiating the paracrine spreading of senescence to other cell types, including DA neurons, and BBB integrity is eventually impaired allowing for T-cell infiltration, as observed in PD. Specifically, HLA-DR + microglia are present in the case of neurodegeneration as an additional marker of T-cell infiltration. In turn, (D) the MHC-I presentation of α-synuclein as an antigen allows for T-cells present in the midbrain to become activated. These activated T-cells can then act to kill DA neurons as seen in PD. Created with BioRender.com.

Overall, there is evidence of senescence in various cell types of the brain in aging and disease and specifically in the midbrain in PD, yet there is still more research to be done. There is a major gap remaining regarding how the paracrine spreading of senescence between different cell types in the midbrain occurs both in normal aging and PD, and how this may contribute to the pathology of the disease. There is an abundance of microglia and T-cells in the SNpc of PD patients even before neurodegeneration and symptoms occur. One explanation might be that senescent endothelial cells at the BBB cause impairment of BBB integrity and a SASP that may recruit T-cells into the midbrain. Alternatively, a senescent cell type in the midbrain of PD patients—DA neurons, astrocytes, or even microglia themselves—may spread senescence via the SASP to other cell types, recruit active microglia, and, in conjunction with decreased BBB permeability, recruit T-cells to actively kill midbrain DA neurons. In either case, some senescent cell type(s) in the SNpc of PD patients would trigger local inflammation and attract immune cells that actively remove the senescent DA neurons (Figure 1). Accordingly, PD may be looked at as a senescence disorder, with senescence in the midbrain and/or at the BBB activating an immune response leading to DA neuron death in the SNpc. Additional support for this hypothesis comes from evidence demonstrating commonalities between triggers of senescence and features of PD. Mutations in *PINK1* and *PARKIN*, which are involved in mitochondrial turnover, are causative for PD (Kitada et al., 1998; Valente et al., 2004) and mitochondrial impairment triggers senescence. Additionally, α-synuclein, a hallmark protein aggregate seen in PD, induces DNA damage and cellular senescence (Yoon et al., 2022). In line with this, lysosomal dysfunction is central to PD pathology (Wallings et al., 2019) and can trigger cellular senescence (Tai et al., 2017). An interesting future area of study would be the relationship between senescence and PD, as it is clear that PD-linked mutant genes are capable of causing senescence and that senescence itself plays a role in PD neuropathology. Given this plethora of evidence, it may be possible to make use of senolytic drugs which target and clear senescent cells as a potential therapeutic intervention for PD. In theory, if senescent cells can be cleared in prodromal PD patients, it may be possible to ameliorate local inflammation and consequently lessen the extent of DA neuron death, thereby preventing PD development or slowing disease progression. In fact, clinical trials for AD using senolytics are currently ongoing, an approach that would be highly promising for PD as well.

## Author Contributions

TR and MR outlined and wrote the manuscript. Both authors contributed to the article and approved the submitted version.

## Conflict of Interest

The authors declare that the research was conducted in the absence of any commercial or financial relationships that could be construed as a potential conflict of interest.

## Publisher’s Note

All claims expressed in this article are solely those of the authors and do not necessarily represent those of their affiliated organizations, or those of the publisher, the editors and the reviewers. Any product that may be evaluated in this article, or claim that may be made by its manufacturer, is not guaranteed or endorsed by the publisher.
